# Long-term iron supplementation combined with vitamin B6 enhances maximal oxygen uptake and promotes skeletal muscle-specific mitochondrial biogenesis in rats

**DOI:** 10.3389/fnut.2023.1335187

**Published:** 2024-01-15

**Authors:** Lei Zhou, Soroosh Mozaffaritabar, Attila Kolonics, Takuji Kawamura, Atsuko Koike, Johanna Kéringer, Yaodong Gu, Roman Karabanov, Zsolt Radák

**Affiliations:** ^1^Research Institute of Molecular Exercise Science, Hungarian University of Sport Science, Budapest, Hungary; ^2^Waseda Institute for Sport Sciences, Waseda University, Saitama, Japan; ^3^Department of Life Sciences, Graduate School of Arts and Sciences, The University of Tokyo, Tokyo, Japan; ^4^Faculty of Sports Science, Ningbo University, Ningbo, China; ^5^Synthesit Swiss SA, Genève, Switzerland

**Keywords:** iron supplementation, vitamin B6, VO_2_ max, skeletal muscle, mitochondrial biogenesis, exercise performance

## Abstract

**Introduction:**

Iron is an essential micronutrient that plays a crucial role in various biological processes. Previous studies have shown that iron supplementation is related to exercise performance and endurance capacity improvements. However, the underlying mechanisms responsible for these effects are not well understood. Recent studies have suggested the beneficial impact of iron supplementation on mitochondrial function and its ability to rescue mitochondrial function under adverse stress *in vitro* and rodents. Based on current knowledge, our study aimed to investigate whether the changes in exercise performance resulting from iron supplementation are associated with its effect on mitochondrial function.

**Methods:**

In this study, we orally administered an iron-based supplement to rats for 30 consecutive days at a dosage of 0.66 mg iron/kg body weight and vitamin B6 at a dosage of 0.46 mg/kg.

**Results:**

Our findings reveal that long-term iron supplementation, in combination with vitamin B6, led to less body weight gained and increased VO_2_ max in rats. Besides, the treatment substantially increased Complex I- and Complex II-driven ATP production in intact mitochondria isolated from gastrocnemius and cerebellum. However, the treatment did not change basal and succinate-induced ROS production in mitochondria from the cerebellum and skeletal muscle. Furthermore, the iron intervention significantly upregulated several skeletal muscle mitochondrial biogenesis and metabolism-related biomarkers, including PGC-1α, SIRT1, NRF-2, SDHA, HSL, MTOR, and LON-P. However, it did not affect the muscular protein expression of SIRT3, FNDC5, LDH, FIS1, MFN1, eNOS, and nNOS. Interestingly, the iron intervention did not exert similar effects on the hippocampus of rats.

**Discussion:**

In conclusion, our study demonstrates that long-term iron supplementation, in combination with vitamin B6, increases VO_2_ max, possibly through its positive role in regulating skeletal muscle-specific mitochondrial biogenesis and energy production in rats.

## 1 Introduction

Iron is classified as a micronutrient. Every cell and organ system in the body requires iron for proper development and subsequent metabolic function. The adult human body contains 3–5 g of iron, with ~70% utilized in hemoglobin of red blood cells (1). Extracellular iron is taken up by cells and transported to the mitochondria, where it is utilized to synthesize cofactors essential for the function of enzymes involved in oxidation–reduction reactions, DNA synthesis and repair, and various other cellular processes (2). Excess iron is stored in the liver and can be mobilized on demand. There are many debates about using iron as a supplement due to its redox reactivity because it might potentially lead to iron overload. Nevertheless, it is very important to highlight that current evidence does not support the idea that dietary intake of inorganic iron, whether from fortified foods or supplements, contributes to the disease burden in the general population (3–5).

Iron is critical to optimal athletic performance because of its role in energy metabolism, oxygen transport, hemoglobin generation and acid–base balance. Iron deficiency is associated with fatigue and decreased exercise capacity (6, 7). Several studies about iron supplementation have shown improvements in physical performance, especially in women with and without iron deficiency (7–10). The physiologic mechanism for these effects may reflect a range of processes. One proposed mechanism is related to hemoglobin concentrations. Hemoglobin is a crucial protein responsible for transporting oxygen in the bloodstream. Iron plays a vital role as an essential component of hemoglobin. Consequently, supplementing with iron may enhance the blood’s ability to carry oxygen, leading to improved tissue oxygenation during physical exercise. Enhanced oxygen delivery to muscles could improve endurance and athletic performance (8). However, it is important to note that this is just one of the potential mechanisms and other factors may also contribute to the improvements in physical performance with iron supplementation. Therefore, additional research is needed to comprehend other underlying mechanisms involved.

Recent studies have suggested the beneficial impact of iron supplementation on mitochondrial function and its ability to rescue mitochondrial function under adverse stress. In *in vitro* studies, one study using zebrafish liver cells discovered that under hypoxia conditions, exogenous iron supplementation inhibits hypoxia-induced cell death, increases reactive oxygen species (ROS) levels, reduces mitochondrial damage, and restores mitochondrial function (11). Besides, another study using yeast cells found that iron supplementation restores the mitochondrial defect of lysosome-impaired mutants (12). Interestingly, one *in vitro* study using yeast cells reported that iron supplementation delays aging and extends cellular lifespan by potentiating mitochondrial function and oxidative stress resistance (13). Almost all tricarboxylic acid (TCA) cycle genes were upregulated by iron supplementation in yeast cells (13). In rodent studies, it has been observed that iron supplements can mitigate obesity and hepatic lipid accumulation induced by a high-fat diet. However, it is important to note that despite the iron supplementation, the animals still exhibited an obese condition (14). Furthermore, iron supplementation reduces mitochondrial morphological abnormalities and upregulates gene transcription involved in mitochondrial function and beta-oxidation in the liver and skeletal muscle (14). In human subjects, long-term iron supplementation is associated with a significant increase in mitochondrial DNA (mtDNA) content (15). Connecting all these findings, we propose a hypothesis that the observed improvement in exercise performance induced by iron (7–10) may be attributed to its potential effect on modulating mitochondrial function. Vitamin B6, a critical co-factor in various physiological processes, is introduced into our study protocol due to its known effects on hemoglobin production, red blood cell formation, and iron transportation (16, 17). Beyond its established role in heme synthesis, vitamin B6 has potential implications for mitochondrial function. Several studies have suggested its involvement in processes such as oxidative phosphorylation and the regulation of reactive oxygen species (ROS) (18). We have included vitamin B6 in our supplement group specifically to improve the effectiveness of iron administration. It is reported that iron is converted into heme the form which is usable by the body in the presence of vitamin B6, which is involved in the decarboxylation reactions that occur within the mitochondrial matrix and are in the process of generating heme, an essential component of hemoglobin (19–21). Vitamin B6 deficiency could affect these functions, by reducing the synthesis of heme and cytochrome as well as increasing the level of reactive oxygen species (22). By including vitamin B6 in our supplementation regimen, we target to elucidate potential synergies between iron and vitamin B6 in modulating mitochondrial function. Overall, the present study aims to investigate the association between long-term iron supplementation, in combination with vitamin B6, and exercise performance, with a specific focus on exploring the potential role of iron and vitamin B6 in modulating mitochondrial function and VO_2_ max.

## 2 Materials and methods

### 2.1 The grouping

Twenty 8-week-old male Sprague Dawley (SD) rats (Charles-River Laboratories, Budapest, Hungary) were randomly divided into two groups (*n* = 10), including 1. Control group, which received saline as placebo, and 2. Supplement group, which received the iron-based supplement. Throughout the experiment, all rats were kept in a thermoneutral room with a temperature range of 20–26°C. They were maintained on a 12:12 h light–dark photoperiod. The rats had *ad libitum* access to standard rodent food and water. The entire experiment was carried out in the Research Center for Molecular Exercise Science, Hungary University of Sport Science, and approved by the Ethical Committee (TE-KEB/15/2023).

### 2.2 Intervention protocol

The supplement used in this study was purchased from SynthesitTM Swiss SA. The supplement group received the supplement at the iron content-based volume, 0.66 mg; Iron/kg; Body weight for 30 consecutive days, along with vitamin B6 at a dosage of 0.46 mg/kg. The control for the supplement group was orally administered saline at a volume that approximated the average volume administered to the supplement group, around 0.15–0.3 mL per animal. For 0.1 mL of the supplement, the concentration of iron is 0.08 mg and the vitamin b6 is 0.056 mg. The composition of the supplement includes water, iron (as iron citrate), pyridoxine hydrochloride (vitamin B6), peppermint oil, and citric acid. 48 h after the last intervention, animals were anesthetized with an intraperitoneal injection of ketamine (Richter, concentration: 100 mg/mL) /xylazine (Produlab Pharma, concentration: 20 mg/mL) cocktail in a dose of 0.1 mL / 10 g bodyweight and intraperitoneally perfused with heparinized ice-cold saline. The brain and gastrocnemius were quickly removed and weighed, and the hippocampus was dissected, frozen in liquid nitrogen, and stored at −80°C for further analysis. A section of the hippocampus and pre-homogenized gastrocnemius were homogenized in a buffer containing: 137 mM NaCl, 20 mM Tris–HCl pH 8.0, 2% NP 40, 10% glycerol, and protease inhibitors (PMSF, aprotinin, leupeptin, orthovanadate). Protein levels were determined using Bradford techniques.

### 2.3 VO_2_ max measurement

The VO_2_ max measurement was conducted using the same methodology as previously described, and the assessment took place in an infrared-light dark environment (23, 24). Animals were first acclimated to the treadmill for 3 days and rested for 3 days before performing the VO_2_ max test. VO_2_ max was measured for each animal, using three criteria: (i) no change in VO_2_ when speed was increased, (ii) rats no longer kept their position on the treadmill, and (iii) respiratory quotient (RQ = VCO_2_/VO_2_) > 1. During the test, the temperature was set at 22 ± 2°C, and humidity was controlled at around 55 + − 10%. The starting speed was 5 m/min, and the speed increased to 5 m/min every 3 min. The VO_2_ max data was recorded when the animals met one of these three criteria.

### 2.4 Western blotting

Proteins were electrophoresed on 8–12% polyacrylamide SDS-PAGE gels and were transferred onto Polyvinylidene Fluoride membranes (PVDF) membranes. The membranes were subsequently washed, and after blocking, PVDF membranes were incubated at 4°C with antibodies including Alpha-Tubulin (T6199), p-MTOR/MTOR (cst5536,2,983), p-AKT/AKT (cst9271,4,691), nNOS (bd610309), eNOS (ab76198), iNOS (a#13120), SIRT3 (cst#5490), PGC-1α (nbp1-04676), SIRT1 (ab110304), CS (ab96600), SDHA (sc98253), FNDC5 (ab174833), HSL (cst18381s), LDH (sc33781), Visfatin/Nampt (ab45890), Synapsin (cst2312), NRF2 (SC-722), CBS (cs14782), MFN1 (sc50330), FIS1 (sc98900), LON-P (66043-1-Ig). After incubation with primary antibodies, membranes were washed 3×10 min in TBS-Tween-20 (TBS-T) and incubated with horseradish peroxidase (HRP)–conjugated secondary antibodies at 4°C for 1 h. After secondary antibody incubation, membranes were repeatedly washed and developed by HRP reagent (Super Signal West Pico Chemiluminescent Substrate, Thermo Scientific #34080). The visualized bands were quantified by ImageJ software and normalized to alpha-tubulin, which served as an internal control. For all the Phosphorylated proteins, the phosphorylated one is developed at first, and then the same PVDF was striped and repeated for the first antibody of the total protein. The ratio was calculated based on the protein expression from the same PVDF.

### 2.5 Mitochondria separation

Fresh cerebellum and gastronomic tissues were first immersed in ice-cold PBS (Phosphate-Buffered Saline) supplemented with 10 mM EDTA and then minced into small pieces. The tissue samples were then subjected to digestion using 0.05% trypsin for 30 min with gentle shaking. After digestion, the samples were centrifuged at 1000 g for 5 min to separate the cellular components. The resulting pellet was re-suspended in a 10-fold volume of IBm1 buffer (50 mM Tris–HCl, 50 mM KCl, 10 mM EDTA, 0.2% BSA, and 0.067 M Sucrose, pH 7.4) and homogenized. Next, the homogenized pellet was suspended in the least possible volume of IBm2 buffer (10 mM Tris–HCl, 3 mM Tris-EGTA, and 0.25 M Sucrose, pH 7.4). This step was aimed to further concentrate the cellular content while maintaining the required buffer conditions for cellular integrity. To determine the protein concentration in the samples, the Bradford assay was employed.

### 2.6 Mitochondria ROS production

Mitochondria (0.3 mg/mL) were incubated in an experimental buffer containing 10 mM Tris/HCl, 5 mM MgCl2, 2 mM KH2PO4, 20 mM EGTA/Tris, and 250 mM Sucrose, adjusted to pH 7.4. To assess ROS (Reactive Oxygen Species) production, the buffer was supplemented with 1 μM Amplex Red, which emits fluorescence at 584 nm when excited at 560 nm. Additionally, horseradish peroxidase (10 IU) was added to the buffer for the monitoring of H2O2-induced fluorescence, following the method described by Votyakova et al. with minor modifications (25). To initiate the ROS assessment, basal ROS production was measured. Subsequently, 10 mM succinate (Suc) and/or 1 μM rotenone (Rot) were sequentially added to the buffer. The addition of succinate as a substrate enhances ROS production due to the reverse electron flux at the level of complex 1. The inhibition of complex 1 by rotenone allows estimation of ROS production related to the forward electron flux and the decrease linked to the reverse electron flux (26). To calibrate the H2O2 production, a known amount of H2O2 was added to the buffer. Fluorimetric assays were carried out at 30°C using a Fluorskan Ascent FL fluorimeter on 96-well plates. Each measurement point was performed in triplicate to ensure accuracy and reproducibility of the results.

### 2.7 Mitochondria ATP production

ATP production rates were determined using a luciferin/luciferase assay, as previously described (27), with some modifications. The measurement of mitochondrial ATP synthesis was conducted in intact mitochondria at a concentration of 0.3 mg/mL in buffer A (10 mM KCl, 25 mM Tris–HCl, 2 mM EDTA, 0.1% BSA, 10 mM KH3PO4, 0.1 mM MgCl2, pH 7.4). The measurements were performed at 30°C using a 96-well luminescent plate reader (Fluorskan Ascent FL, Thermo Scientific) with white-walled, white-based 96-well plates. To initiate the assay, 50 μL aliquots of intact mitochondria were incubated for 5 min with 50 μL of a 25x diluted FLAAM luciferase/luciferin reagent (Sigma, St. Louis, MO). Two different sets of reactions were performed to measure ATP production through specific mitochondrial complexes: For measuring ATP production via complex-I: The reaction was carried out with 5 mM malate plus 5 mM glutamate. For measuring ATP production via complex-II: The reaction was conducted with 10 mM succinate plus 2 μg/mL rotenone. Both complex-I-driven and complex-II-driven reactions were performed in the presence and absence of 10 μg/mL oligomycin, an ATP synthase inhibitor. Luminescence, which correlates with ATP concentration, was monitored for 5 min before the addition of 0.2 mM ADP (adenosine diphosphate), after which luminescence was monitored for an additional 30 min. The rate of ATP synthesis was found to be linear and dependent on the concentration of mitochondria and substrate. Each data point was measured in triplicate to ensure the accuracy and reproducibility of the results.

### 2.8 Statistical analysis

The statistical analysis was performed using unpaired Student-*T* TEST analysis followed using Graph pad Prism version 9.1 software. Data were reported as mean ± standard deviation (SD). Statistical significance was denoted as *p<0.05.

## 3 Results

### 3.1 Long-term iron and vitamin B6 supplement leads to less body weight gain and elevates VO_2_ max in rats

**Figure 1:**
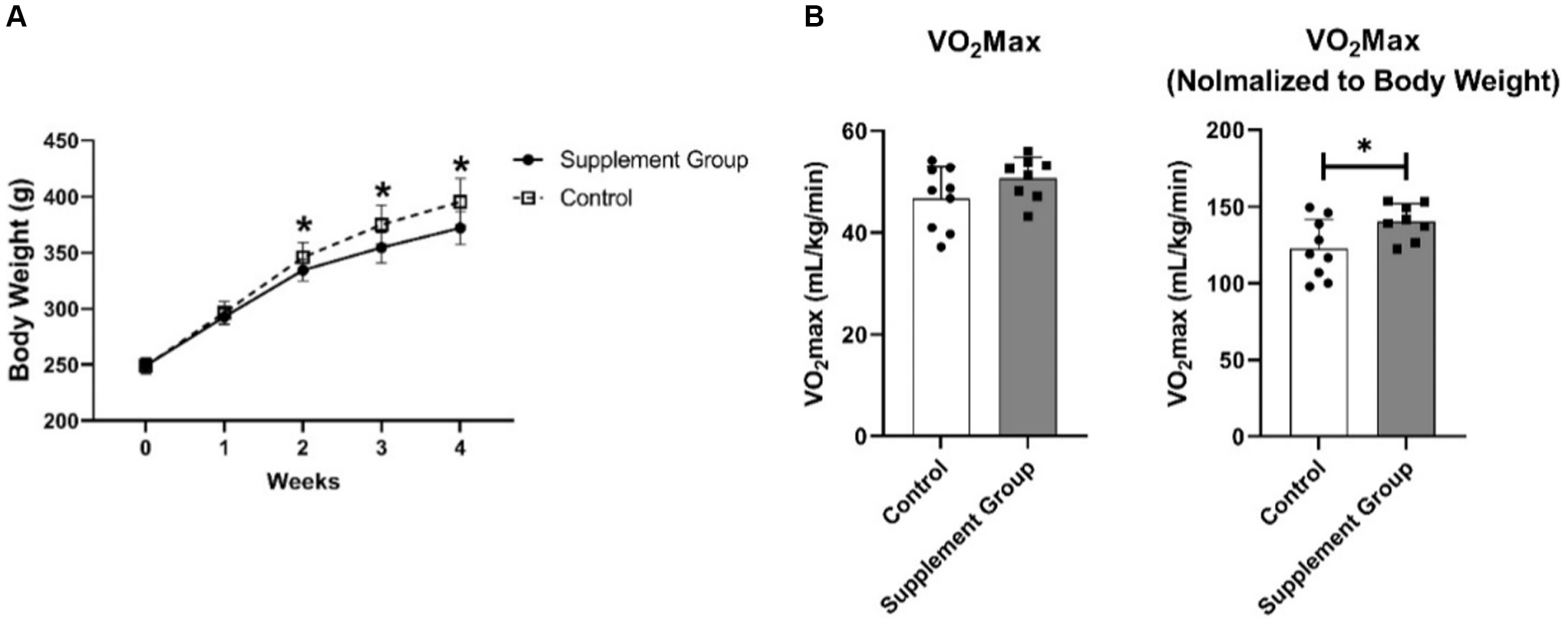
**(A)** The effect of iron and vitamin B6 supplement on rats’ body weight (*n* = 10). **(B)** The effects of long-term iron and vitamin B6 supplement on VO_2_ max (*n* = 8). Data are presented as means ± SD. Statistical significance was denoted as *p<0.05.

As presented in Figure 1A, long-term intake of iron and vitamin B6 supplement led to less body weight gain in rats. The result started to be different (*p* = 0.04) after the second week of intervention and continued to be more prominent in the third (*p* = 0.01) and the fourth (*p* = 0.009) weeks. Furthermore, after 30 days of intervention, the VO_2_ max increased (*p* = 0.038) in the supplement group (Figure 1B). It is important to mention that we normalized the original VO_2_ max data to body weight. The original VO_2_ max has an increasing tendency (*p* = 0.15) and the lower body weight led to the normalized VO_2_ max to be significant.

### 3.2 Long-term iron and vitamin B6 supplement improves mitochondrial function of cerebellum and gastrocnemius in rats

**Figure 2:**
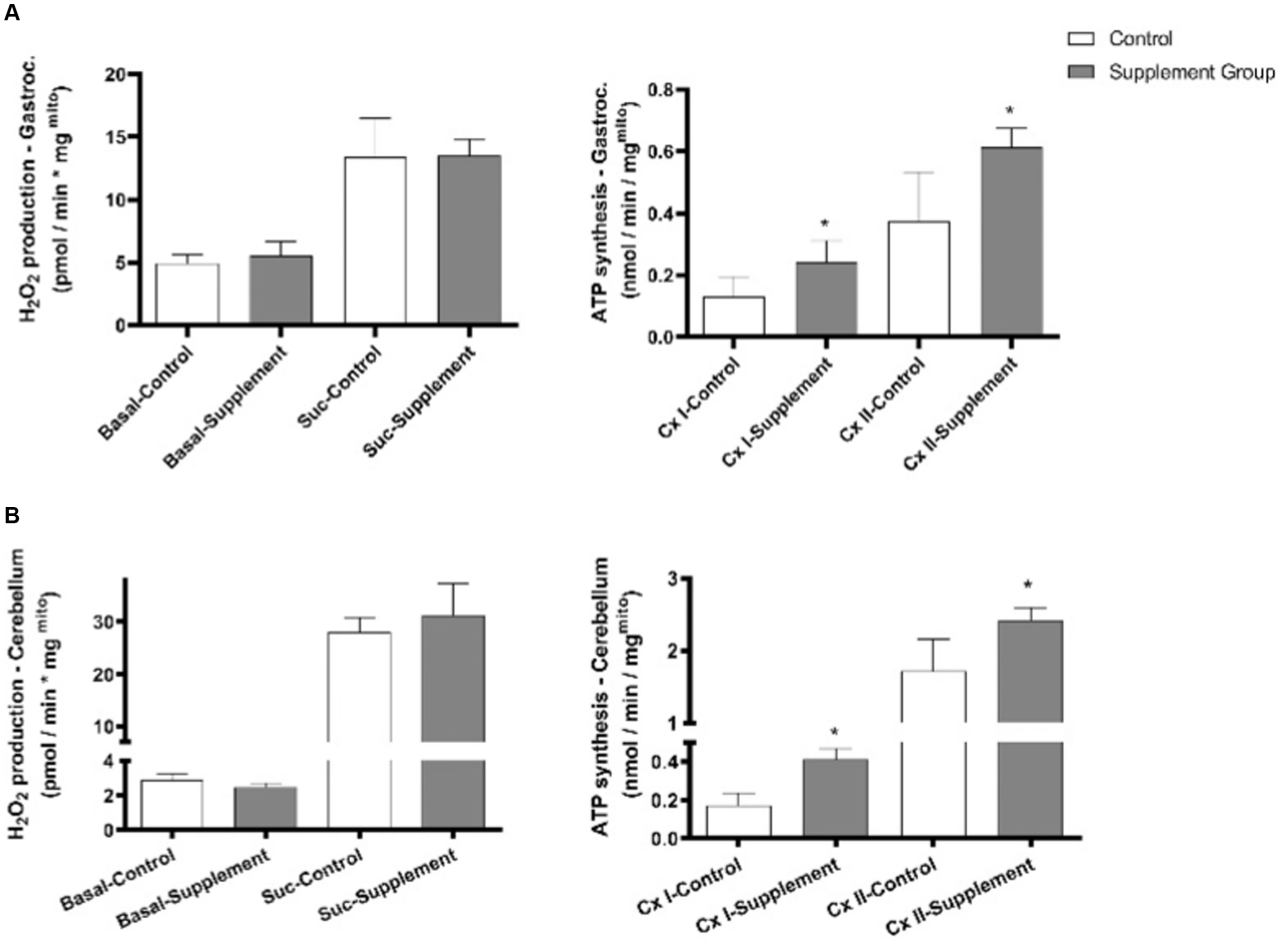
**(A)** Effects of iron and vitamin B6 supplementation on mitochondrial ROS production and ATP production in rat gastrocnemius (*n* = 5). **(B)** Effects of iron and vitamin B6 supplementation on mitochondria ROS production and ATP production in rat cerebellum (*n* = 5). In case of ATP production, malate and glutamate were added to drive Complex I, and succinate and rotenone were added to drive Complex II. In case of ROS production, a distinct comparison is made between basal ROS production and ROS production induced by succinate. In the ATP production section, separate comparisons are drawn for complex I and complex II. Data are presented as mean ± SEM (*n* = 5). Statistical significance was denoted as *p<0.05.

As presented in Figure 2, iron and vitamin B6 supplementation had no significant effects on changing the basal ROS production and succinate-induced ROS production in the freshly isolated mitochondrial from rats’ gastrocnemius and cerebellum. On the other hand, iron and vitamin B6 supplementation substantially increased Complex I- and Complex II-driven ATP production in intact mitochondria isolated from the rats’ gastrocnemius and cerebellum.

### 3.3 Long-term iron and vitamin B6 supplement increases mitochondrial biogenesis and metabolism-related biomarkers in the skeletal muscle of rats

**Figure 3:**
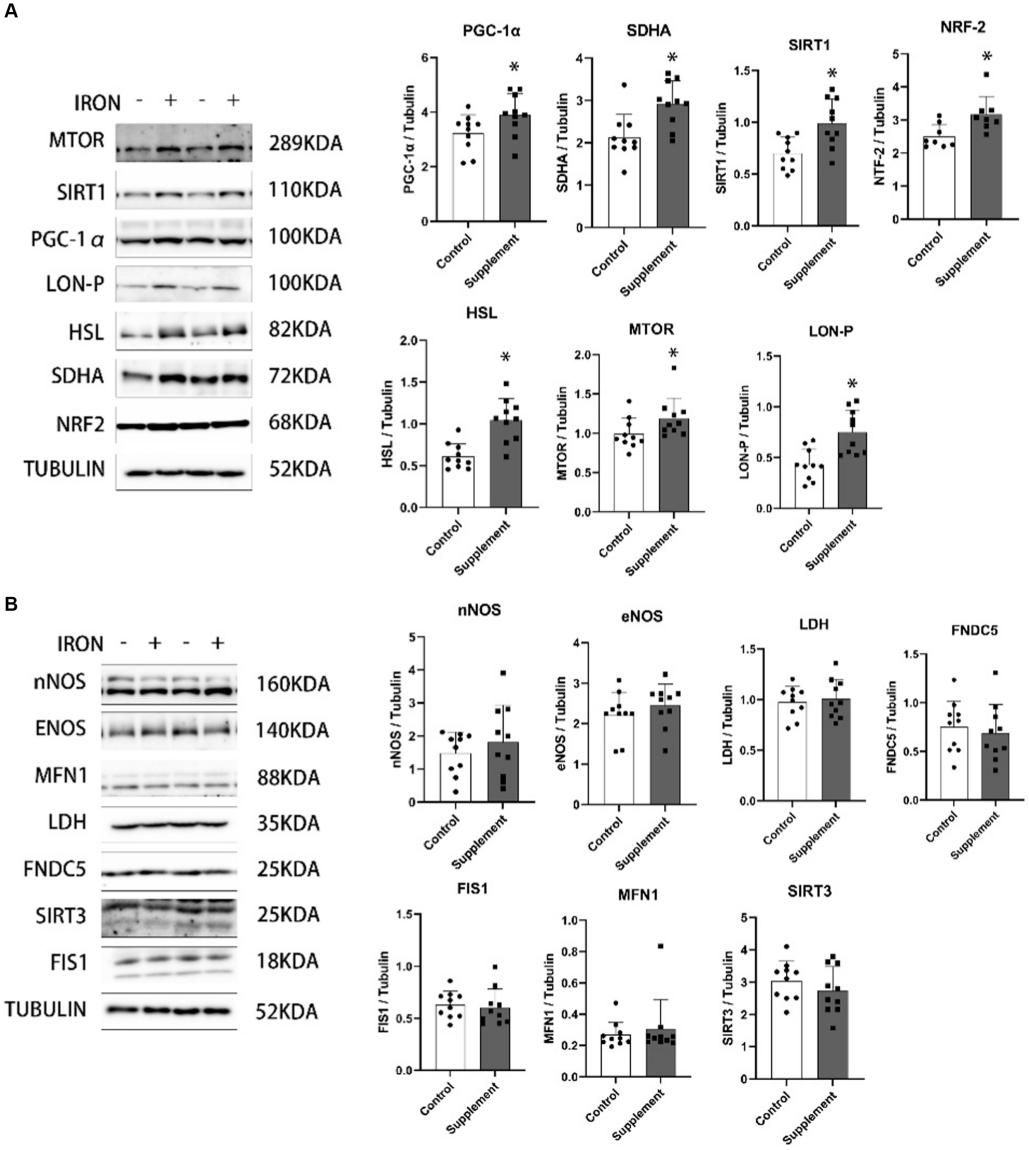
**(A)** The effects of iron and vitamin B6 supplement on mitochondrial biogenesis and metabolism–related biomarkers in skeletal muscle of rats (*n* = 10). **(B)** The effects of iron and vitamin B6 supplement on metabolic and adaptive related proteins in skeletal muscle of rats. Data are presented as means ± SD (*n* = 10). Statistics are calculated by each animal’s data. Statistical significance was denoted as *p<0.05.

As presented in Figure 3A, we observed that after 30 days of iron and vitamin B6 supplementation in rats, the protein levels of peroxisome proliferator-activated receptor gamma coactivator 1 alpha (PGC-1α), sirtuin 1 (SIRT1), nuclear factor erythroid 2-related factor 2 (NRF-2), succinate dehydrogenase complex subunit A (SDHA), hormone-sensitive lipase (HSL), mammalian target of rapamycin (mTOR), and LON protease (LON-P) in skeletal muscle were significantly higher compared to the control group. On the other hand, the protein expression of neuronal nitric oxide synthase (nNOS), endothelial nitric oxide synthase (eNOS), lactate dehydrogenase (LDH), fibronectin type III domain containing protein 5 (FNDC5), mitochondrial fission 1 protein (FIS1), mitofusin 1 (MFN1), and sirtuin 3 (SIRT3) did not show any significant changes after the intervention (Figure 3B).

### 3.4 Long-term iron and vitamin B6 supplement does not influence biomarkers of mitochondrial biogenesis, metabolism, and adaptive capacity in the hippocampus of rats

**Figure 4:**
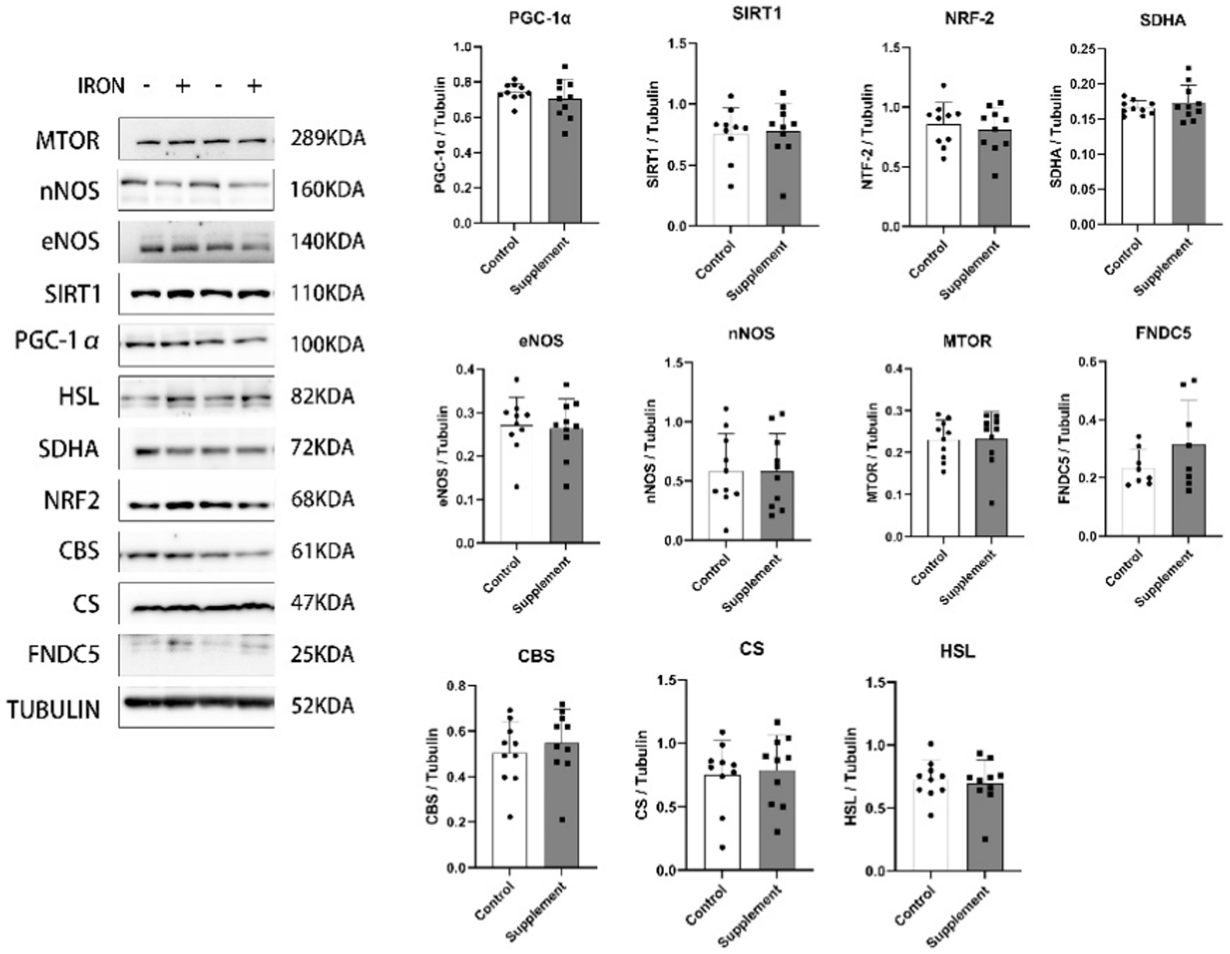
The effects of iron and vitamin B6 supplement on mitochondrial biogenesis biomarkers, metabolic and adaptive capacity-related proteins in the hippocampus of rats (*n* = 10). Data are presented as means ± SD. Statistics are calculated by each animal’s data. Statistical significance was denoted as *p<0.05.

As presented in Figure 4. our data showed that the mitochondrial biogenesis effect of long-term iron and vitamin B6 supplement is tissue specific. 30 days of iron and vitamin B6 intervention did not exert similar effects on the hippocampus. After the intervention, the protein expression of PGC-1α, SIRT1, SDHA, NRF2, eNOS, nNOS, HSL, MTOR, FNDC5, cystathionine beta-synthase (CBS), and citrate synthase (CS) remained unchanged in the hippocampus of rats.

## 4 Discussion

The primary objective of this study was to explore the relationship between iron supplementation combined with vitamin B6, VO_2_ max, and mitochondrial function in skeletal muscle and the brain of rodents. Previous research has shown contradictory findings regarding the effects of iron supplementation on exercise performance and endurance capacity (8, 10). To address this inconsistency, we used an iron supplement containing vitamin B6 in our study. The reason for the inclusion of vitamin B6 aimed to enhance the efficacy of iron administration due to the crucial role of this vitamin in hemoglobin synthesis, red blood cell formation, and iron transportation (16, 17). Additionally, research has demonstrated that vitamin B6 facilitates the conversion of iron into heme, the biologically active form of iron involved in the body’s biochemical reactions, including hemoglobin synthesis within the mitochondrial matrix during decarboxylation reactions (19–21). Moreover, the specific underlying mechanisms responsible for the effects of iron supplementation on exercise performance have not been thoroughly investigated. Earlier studies have suggested that the beneficial effects of iron supplement are primarily attributed to its impact on hemoglobin levels and iron storage in the body (8). Recent studies suggested that iron supplementation may benefit mitochondrial function and adaptive capacity under adverse stress (11–15). Therefore, in this study we used rats to investigate the potential effects of iron combined with vitamin B6 supplementation on VO_2_ max, and mitochondrial biogenesis.

This experiment orally administered an iron-based supplement to rats for a duration of 30 consecutive days. The dosage used was 0.66 mg iron/kg body weight, combined with vitamin B6 at a dosage of 0.46 mg/kg. The results of this study revealed several important findings. Firstly, the long-term iron-based supplementation led to less body weight gain in the rats. One previous study discovered that iron supplementation can alleviate high-fat-diet-induced obesity (14). Here our results showed that long-term iron-based supplement leads to less body weight gained of healthy animals under normal diet. However, further investigations are required to elucidate the underlying mechanisms involved. Secondly, the rats exhibited an increase in VO_2_ max, which is a measurement of maximal oxygen uptake and an indicator of aerobic capacity. Our result is in line with previous studies showing that long-term iron supplements could elevate the VO_2_ max (8–10). It is crucial to note that the original VO_2_ max exhibits an increasing tendency, with a *p*-value of 0.15, indicating non-significance. However, when utilizing body weight for normalization, significant increases were observed. It’s important to acknowledge that lean mass is typically more correlated with VO_2_ max (28), but our study did not directly measure lean mass. Instead, body weight was employed for normalization purposes. This deviation in measurement has limitation to the study.

To investigate the potential mechanisms responsible for the observed enhancements in VO_2_ max and reductions in body weight gained, we analyzed isolated intact mitochondrial function from the gastrocnemius muscle and assessed various biomarkers related to skeletal muscle mitochondrial biogenesis and metabolism. The results indicate that iron and vitamin B6 supplementation significantly increased complex I- and complex II-driven ATP production in intact mitochondria isolated from the rat gastrocnemius and substantially upregulated the expression of several biomarkers directly or indirectly involved in mitochondrial biogenesis pathways, including PGC-1α, SIRT1, SDHA, NRF2, HSL, mTOR, and LON-P. Notably, PGC-1α protein plays a pivotal role in regulating mitochondrial biogenesis and skeletal muscle development (29). SIRT1 has emerged as an active regulator of muscle repair, hypertrophy, and energy metabolism, and its actions on PGC-1α could drive mitochondrial metabolic adaptation (30, 31). The observed elevation of both PGC-1α and SIRT1 proteins suggests a coordinated response favoring mitochondrial adaptation, potentially facilitating improved VO_2_ max. Furthermore, our investigation demonstrated an increase in NRF2, a pivotal transcription factor responsible for complete exercise-induced mitochondrial adaptation in skeletal muscles (32). SDHA, a key enzyme in the mitochondrial respiratory chain, was found to play a critical role in cellular respiration and mitochondrial biogenesis (33). In addition, HSL was identified to regulate lipid metabolism in skeletal muscle (34), while mTOR was shown to be critical in controlling skeletal muscle mass through its influence on protein synthesis and energy metabolism (35). Moreover, LON-P was found to have a crucial role in maintaining mitochondrial homeostasis and biogenesis (36). These findings suggest that the observed improvement in VO_2_ max may be attributed partially to the positive effects of iron and vitamin B6 on regulating skeletal muscle mitochondrial biogenesis and energy production biomarkers. Overall, this study suggests that the supplementation of iron and vitamin B6 positively influences mitochondrial function and might have beneficial effects on skeletal muscle health and exercise performance. It is important to note that in this study, we did not measure the fat content and lean body mass. Therefore, we can only hypothesize that the reduction in body weight gain might be attributed to a potential decrease in fat content due to the upregulation of energy production and metabolism-related proteins. However, to ascertain the specific mechanism responsible for the observed changes, further studies are required. Recent studies conducted *in vitro*, rodents, and humans have proposed iron’s beneficial effects on mitochondrial functions. *In vitro* studies demonstrate that iron supplementation can lead to the upregulation of nearly all genes related to the TCA cycle (13). In rodent models, iron supplementation has been shown to upregulate gene transcription involved in mitochondrial function and beta-oxidation in both the liver and skeletal muscle (14). Furthermore, in humans, iron supplementation has been associated with a significant increase in mtDNA content (15). Here, our results further tested the mitochondrial biogenesis effects of iron in the skeletal muscle of rodents. Additionally, our findings revealed that iron supplementation had no significant effect on both basal and succinate-induced ROS production in the freshly isolated mitochondria from rat’s gastrocnemius. Moreover, iron supplementation did not influence the protein expression of nNOS, eNOS, LDH, FNDC5, FIS1, MFN1, and SIRT3.

Interestingly, we did not observe similar effects of iron intervention on the hippocampus of the rats. The protein expression of PGC-1α, SIRT1, SDHA, NRF2, eNOS, nNOS, HSL, MTOR, FNDC5, CBS, and CS did not change in the hippocampus following the intervention. This suggests that the impact of supplementation on mitochondrial function might be specific to skeletal muscle and may not extend to other tissues or organs. Previous studies have indicated that appropriate iron supplementation during developmental and rapidly growing periods of rodents improves memory performance (37). On the other hand, some studies reported that iron overload may be associated with neurodegenerative diseases (38). Our results showed that 0.66 mg/kg dosage of iron supplement in combination with vitamin B6 does not influence the biomarkers of mitochondrial biogenesis, metabolism, and adaptive capacity in the hippocampus of rats. Further studies are needed to explore the dose-dependent effect of iron supplements. In addition to the hippocampus, our result shows that iron and vitamin B6 treatment substantially increased complex I- and complex II-driven ATP production in intact mitochondria isolated from the cerebellum but resulted in no significant changes in basal and succinate-induced ROS production. Previous study has reported that excess iron may induce oxidative stress and thereby cause tissue damage (3). Here, our study confirmed that iron supplement at a dosage of 0.66 mg/kg in combination with vitamin B6 does not influence the ROS production in the cerebellum and skeletal muscle. Further investigations are warranted to explore the potential dosage-dependent effects of iron supplements on overall health.

## 5 Conclusion

In conclusion, this study contributes to understanding the relationship between iron supplementation, exercise performance, and mitochondrial function. The findings suggest that long-term iron-based supplementation in combination with vitamin B6 may improve exercise performance, as indicated by the increased VO_2_ max in rats. These improvements could be attributed to its positive role in regulating skeletal muscle mitochondrial biogenesis, as evidenced by the upregulation of key biomarkers and energy production. However, it is important to note that these findings are based on animal research, and further studies are required to validate these findings in human subjects. Understanding the mechanisms by which iron affects mitochondrial function and VO_2_ max can have implications for the development of strategies to optimize athletic performance and improve overall health and well-being. Furthermore, we supplemented iron with vitamin B6 together; thus, this study has limitations in that we cannot conclude the specific impact of each component. The observed beneficial effects are likely a result of their synergistic action. Additionally, it is essential to consider the dosage of iron and vitamin B6 for supplementation, as it may play a crucial role in their effects. However, this study did not explore the impact of different dosages on the outcomes. The dosages utilized in this study were 0.66 mg/kg for iron and 0.46 mg/kg for vitamin B6, which did not lead to an increase in ROS in the mitochondria of the cerebellum and skeletal muscle. Moreover, this dosage induced mitochondrial biogenesis in the skeletal muscle only. Future research is warranted to validate the optimal dosages that produce the desired outcomes without causing potential adverse effects. As with any supplementation, it is better to consult a healthcare professional or sports nutrition expert to determine the appropriate approach based on an individual’s specific needs and circumstances.

## Data availability statement

The raw data supporting the conclusions of this article will be made available by the authors, without undue reservation.

## Ethics statement

The animal study was approved by Hungary University of Sport Science (TE-KEB/15/2023). The study was conducted in accordance with the local legislation and institutional requirements.

## Author contributions

LZ: Conceptualization, Data curation, Methodology, Project administration, Software, Writing – original draft, Writing – review & editing. SM: Conceptualization, Data curation, Methodology, Project administration, Writing – original draft, Writing – review & editing. AttK: Methodology, Writing – review & editing. TK: Methodology, Writing – review & editing. AtsK: Methodology, Writing – review & editing. JK: Methodology, Writing – review & editing. YG: Visualization, Writing – review & editing. RK: Resources, Writing –review & editing. ZR: Conceptualization, Funding acquisition, Investigation, Visualization, Writing – review & editing, Writing – original draft.
